# Surface guidance compared with ultrasound-based monitoring and diaphragm position in cone-beam computed tomography during abdominal stereotactic radiotherapy in breath-hold

**DOI:** 10.1016/j.phro.2023.100455

**Published:** 2023-06-05

**Authors:** Lena Kaestner, Lara Streb, Svetlana Hetjens, Daniel Buergy, Dwi S.K. Sihono, Jens Fleckenstein, Iris Kalisch, Miriam Eckl, Frank A. Giordano, Frank Lohr, Florian Stieler, Judit Boda-Heggemann

**Affiliations:** a University Medical Center Mannheim, Department of Radiation Oncology, University of Heidelberg, Theodor-Kutzer-Ufer 1-3, 68167 Mannheim, Germany; b University Medical Center Mannheim, Department of Medical Statistics and Biomathematics, University of Heidelberg, Theodor-Kutzer-Ufer 1-3, 68167 Mannheim, Germany; c Departemen Fisika, FMIPA, Universitas Indonesia, Depok 16424, Indonesia; d Struttura Complessa di Radioterapia, Dipartimento di Oncologia, Az. Ospedaliero-Universitaria di Modena, Largo del Pozzo 71, 41122 Modena, Italy

**Keywords:** SBRT, DIBH (deep-inspiration-breath-hold), Ultrasound-based monitoring, SGRT (surface-guided radiotherapy)

## Abstract

**Background and purpose:**

Spirometry induced deep-inspiration-breath-hold (DIBH) reduces intrafractional motion during upper abdominal stereotactic body radiotherapy (SBRT). The aim of this prospective study was to evaluate whether surface scanning (SGRT) is an adequate surrogate for monitoring residual internal motion during DIBH. Residual motion detected by SGRT was compared with experimental 4D-ultrasound (US) and an internal motion detection benchmark (diaphragm-dome-position in kV cone-beam computed tomography (CBCT) projections).

**Materials and methods:**

Intrafractional monitoring was performed with SGRT and US in 460 DIBHs of 12 patients. Residual motion detected by all modalities (SGRT (anterior-posterior (AP)), US (AP, craniocaudal (CC)) and CBCT (CC)) was analyzed. Agreement analysis included Wilcoxon signed rank test, Maloney and Rastogi’s test, Pearson’s correlation coefficient (PCC) and interclass correlation coefficient (ICC).

**Results:**

Interquartile range was 0.7 mm (US(AP)), 0.8 mm (US(CC)), 0.9 mm (SGRT) and 0.8 mm (CBCT). SGRT(AP) vs. CBCT(CC) and US(CC) vs. CBCT(CC) showed comparable agreement (PCCs 0.53 and 0.52, ICCs 0.51 and 0.49) with slightly higher precision of CBCT(CC). Most agreement was observed for SGRT(AP) vs. US(AP) with largest PCC (0.61) and ICC (0.60), least agreement for SGRT(AP) vs. US(CC) with smallest PCC (0.44) and ICC (0.42).

**Conclusions:**

Residual motion detected during spirometry induced DIBH is small. SGRT alone is no sufficient surrogate for residual internal motion in all patients as some high velocity motion could not be detected. Observed patient-specific residual errors may require individualized PTV-margins.

## Highlights

• Residual motion during spirometry induced deep-inspiration-breath-hold is small.
• Surface tracking alone is no sufficient surrogate for internal motion in all patients.
• Real-time 3D soft-tissue monitoring assures maximum security.
• Observed patient-specific residual errors may require individualised safety margins.

## Graphical abstract

**Figure:**
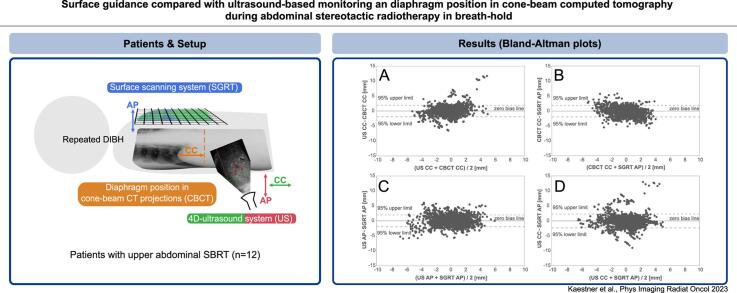

## 1 Introduction

Stereotactic body radiotherapy (SBRT) is a non-invasive treatment option for local control of abdominal oligometastases [1], [2], [3], [4], [5], [6], [7]. Delivery chain for SBRT requires maximum precision during planning and beam delivery [8]. Between treatment fractions high interfractional repositioning accuracy is already achievable with, e.g., daily cone-beam computed tomography (CBCT)-based image-guided radiotherapy and deep-inspiration-breath-hold (DIBH) [9], [10], [11], [12]. Residual motion during DIBH can be detected via intrafractional monitoring with magnetic resonance imaging (MRI) linear accelerator [13], [14], 4D-ultrasound (US) [15], [16] or surface-guided radiotherapy (SGRT) [17], [18], [19], [20], [21] during repositioning and beam application. Alternatively, offline determination of surrogate structures in CBCT such as fiducial markers or the diaphragm-dome can help to estimate possible ranges of residual motion [22], [23], [24]. All in all, the implementation of intrafractional motion management allows further planning target volume (PTV)-margin reduction, dose escalation and toxicity reduction [25], [26], [27].

US-based monitoring for radiotherapy of prostate cancer [28], [29], [30] is already widely used and enables online 4D inter- and intrafractional detection of internal motion. For abdominal scanning, a clinical workflow has been established with an experimental US-system [16], [31]. As complexity of radiotherapy increases with the need for precise soft-tissue imaging, US-based monitoring is a promising method with possible advantages compared to MRI in terms of imaging rates and compatibility with simple linear accelerator systems [32]. SGRT-systems have been implemented for breast radiotherapy [33] and during abdominal/thoracic SBRT [18], [19], [20]. However, their accuracy as surrogate for internal motion during abdominal DIBH-SBRT is still controversial and previous literature lacks comparison with internal monitoring, e.g., an US-system [18], [23], [34], [35], [36], [37], [38].

The aim of this prospective study was to evaluate whether SGRT is an adequate surrogate for monitoring residual intrafractional internal motion during upper abdominal DIBH-SBRT. Detected residual motion of the SGRT-system was compared with detected internal residual motion of a 4D-US and the kV-projections of the diaphragm-dome (DD) in DIBH-CBCT as internal motion detection benchmark.

## 2 Materials and methods

### 2.1 Patients and monitoring systems

Data of 460 DIBHs in 74 fractions of 12 patients with abdominal SBRT were acquired in a framework of a prospective study after informed consent and local Institutional Review Board agreement (2014-413M-MA-§ 23b MPG). An overview of patients’ characteristics, tumor location and DIBH properties are shown in supplementary Table S1.

The optical SGRT-system Catalyst HD (C-RAD, Sweden) provides the options for interfractional repositioning and breath-hold (BH) gating during imaging and beam delivery [39] with a position and motion detection accuracy below 1 mm (https://c-rad.com/products/catalyst-hd/). Clarity Autoscan/Anticosti™ (Elekta AB, Sweden) is an experimental, commercially not available 4D-US for abdominal scanning, interfractional repositioning and motion monitoring. It is derived from the Autoscan system for prostate repositioning and perineal prostate monitoring [30], [40]. Briefly, the US consists of a computer-cart and a 4D-US probe mounted on a flexible arm (CIVCO, USA) and detected via ceiling-mounted infrared cameras (Polaris, NDI, Canada). For upper abdominal targets the probe was placed on the right medioaxillary line during CBCT acquisition and treatment delivery [41]. Application of the US probe was performed by experienced staff only to avoid inconsistent image quality and thus reduced accuracy [42]. The US probe scans the pre-defined target structure to create a 3D reconstructed volume with a motor-driven sweeping motion (framerate: 45 Hz). Daily quality assurance has a tolerance of 1 mm, the accuracy of the system based on readings in a 4D-phantom is 1.4 ± 1.6 mm for a scanning range of 30°. The monitoring software is based on a prostate tracking-model applied for faster moving targets [16], [31], [43].

### 2.2 Treatment algorithm/workflow

Planning-CT (Brilliance Big Bore, Philips, Netherlands) with a slice thickness of 3 mm was acquired in computer-controlled DIBH with a predefined volume for 15–20 s with ABC® (active breathing coordinator, Elekta AB, intrafractional targeting error <2 mm [44], [45]). At the point of time when the study was conducted, we solely used the ABC®-system for DIBH-guidance during abdominal SBRT. A marker was affixed to the patient surface inferior of the costal arch, defining the gating point and the DIBH window (±2 mm) in relation to the isocenter for SGRT. Subsequently, a reference US dataset was acquired in DIBH and a suitable echogenic monitoring structure (Gross Tumor Volume [GTV] itself or a nearby surrogate vessel [46]) was contoured offline.

After positioning with laser the actual position of the US target was detected and the table-shift was performed (all steps in ABC®-controlled DIBH with a gating interface (Response, Elekta AB)) [43]. Final interfractional repositioning was performed after kV-CBCT (x-ray volume imaging, XVI, Elekta AB) in DIBH only (median 6.3 DIBHs/CBCT). CBCT reconstructions had a resolution of 1 mm (medium) and 0.5 mm (high) with the XVI panel acquiring images with 512 × 512 pixels for a panel size of 41x41 cm (isocenter ∼26 × 26 cm) [47].

During DIBH-CBCT acquisition as well as beam delivery residual motion during DIBH was monitored with SGRT and US. An illustration of the experimental setup can be found in supplementary Fig. S1. As the 4D-US is not commercially available and a theoretically ideal gold standard such as online 4D-MRI was not available we additionally analyzed the kV-projections, and with that the craniocaudal (CC) motion of the diaphragm-dome in CBCT, as a surrogate to acquire as much information on internal motion as possible. The surface motion of the abdominal wall was registered and defined as the anterior-posterior (AP) deviation of the predefined gating point. SGRT delivered a numeric AP surface mismatch value between the actual and reference position every 100  ms. The internal motion of the US target structure was recorded in intervals of 0.5 to 1.0 s in CC, AP and left–right direction.

### 2.3 Monitoring efficacy

Out of 460 performed DIBHs US data was available for further analysis in 336 DIBHs (7535 readings), SGRT data in 325 DIBHs (7244 readings) and CBCT data in 294 DIBHs (6483 readings). In 283 DIBHs data of all motion detection methods could be evaluated. In the excluded DIBHs either monitoring of the target structure/surface was not possible in US/SGRT or the DD was not visible in some CBCT projection angles.

### 2.4 Data transfer and offline statistical analysis

The residual motion of the diaphragm-dome during DIBH-CBCT was determined offline as published previously [24]. Briefly, the CC position of the highest point of the DD during DIBH-CBCT (every 5th projection) was compared with the CC position of a Ball-Bearing phantom (Elekta AB) during CBCT placed at the DD position in planning-CT. To account for projection magnification of the resulting residual motion of the DD an angle-dependent reduction factor was used. For residual motion determination of the DD in kV-projections an error of 2.2 mm corresponding to the CT slice thickness was estimated.

After acquisition of the raw data, timestamps of CBCT and SGRT were adjusted to the US timestamp. Coordinate systems were unified with positive values indicating motion in caudal/anterior direction. For each DIBH readings of US (mean 22 readings/DIBH), SGRT (mean 246 readings/DIBH) and CBCT (mean 19 readings/DIBH) were numerically equated with US readings and normalized to the mean value defined as baseline position. Intrafractional residual motion was defined as the absolute magnitude of the deviation of one reading from the mean value of the DIBH to which this reading belongs.

Data were analyzed with SAS (Version 9.4), JMP (SAS Institute, USA) and Microsoft Excel (Microsoft Corporation, USA). Basic statistical parameters on the detected residual motion of each method were calculated. Agreement between the different motion detection methods (SGRT vs. CBCT, US(CC) vs. CBCT, SGRT vs. US(AP), SGRT vs. US(CC)) was assessed in order to compare residual motion data and assess reliability: Since the requirements for a paired *t*-test were not met (differences not normally distributed), Wilcoxon signed rank test was performed to provide evidence of whether the measurement methods differed systematically [48]. Maloney and Rastogi’s test and Bland-Altman plot were chosen to analyze differences in precision [49], [50]. Correlation analysis was conducted using Pearson’s correlation coefficient (PCC) and interclass correlation coefficient (ICC) [51], [52]. For detailed analysis per DIBH and patient, a PCC was determined for DIBHs with an intrafractional residual motion ≥2 mm in at least one analyzed motion detection method. PCC was considered significantly positive if ≥0.5 (p < 0.05) and significantly negative if ≤−0.5 (p < 0.05). Residual motion <2 mm per DIBH in both compared motion detection methods was considered as ideal “quasi-static” situation, therefore no PCC was calculated for those DIBHs. Individual residual motion curves of each patient were visually evaluated.

## 3 Results

### 3.1 Magnitude of residual motion during DIBH

**Fig. 1:**
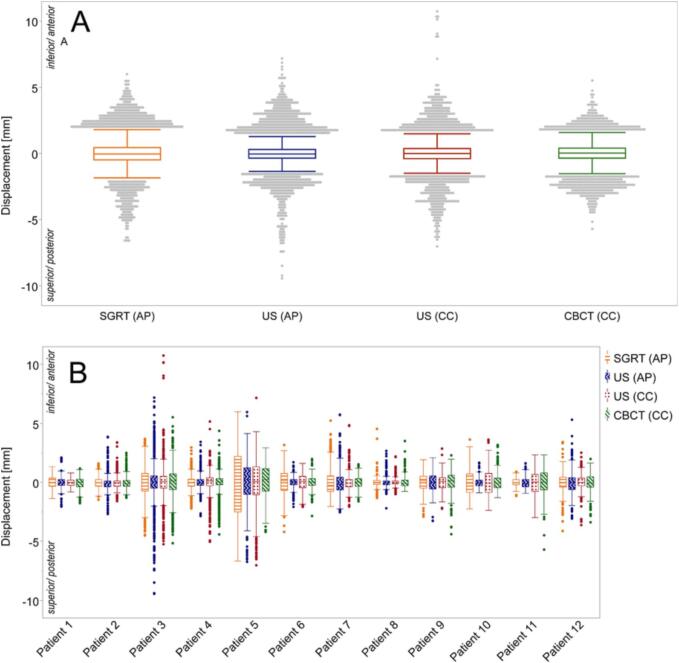
Box plot diagrams of residual motion for all analyzed motion detection methods. Boxes illustrate the interquartile range; whiskers show minimum and maximum data within 1.5 times the interquartile range. Outliers are illustrated as single readings and are defined as data outside 1.5 times the interquartile range. Inferior and anterior directions are positive, superior and posterior are negative. A) Box plot diagrams with outliers for all available readings. Maximum residual motion is highest in US (CC), IQR is highest in SGRT. B) Box plot diagrams with outliers for each patient. Especially patient 3 and 5 show larger residual motion.

Distribution of all analyzed readings with interquartile ranges (IQR) of 0.7 mm (US(AP)), 0.8 mm (US(CC)), 0.9 mm (SGRT) and 0.8 mm (CBCT) can be found in Fig. 1. Maximum numerical residual motion metrics were lowest in CBCT (5.5 mm caudal and 5.7 mm cranial) and highest in US (7.2 mm anterior/9.5 mm posterior and 10.8 mm caudal/7.0 mm cranial). In SGRT maximum residual motion metrics were 6.0 mm anterior and 6.7 mm posterior. Residual motion was <2 mm in 92% of all readings in both US(AP) and SGRT. In US(CC) 94% and in CBCT 95% of all readings showed residual motion <2 mm.

### 3.2 Agreement analysis

**Fig. 2:**
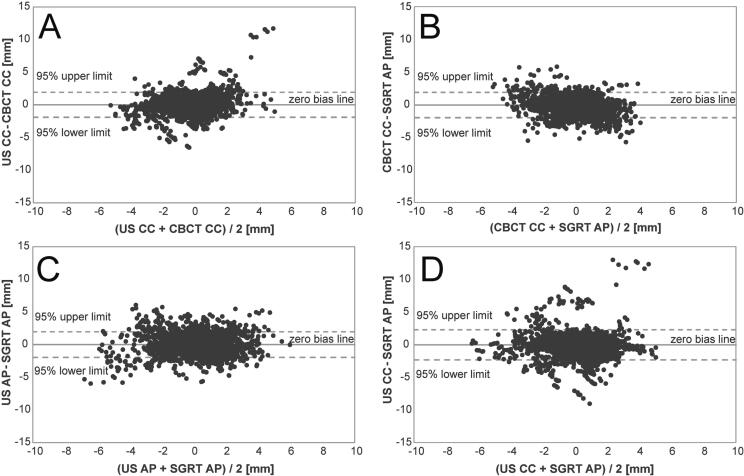
Bland-Altman plots for pairs of motion detection methods with mean value on x-axis and difference on y-axis. Differences between motion detection methods were not normally distributed, as approximation and better visualization 95% confidence intervals and zero bias line are displayed. A) US(CC) vs. CBCT. B) SGRT vs. CBCT. C) SGRT vs. US(AP). D) SGRT vs. US(CC).

SGRT(AP) vs. CBCT(CC) and US(CC) vs. CBCT(CC) showed comparable agreement (PCCs 0.53 and 0.52, ICCs 0.51 and 0.49) with slightly higher precision of CBCT(CC), see Table 1 and Fig. 2. Most agreement was observed for SGRT(AP) vs. US(AP) with largest PCC (0.61) and ICC (0.60), least agreement for SGRT(AP) vs. US(CC) with smallest PCC (0.44) and ICC (0.42).

**Table 1:** Agreement analysis and percentage of correlating DIBHs between analyzed motion detection methods: Most agreement for SGRT vs. US(AP), followed by SGRT vs. CBCT and US(CC) vs. CBCT. Most correlating DIBHs for SGRT vs. US(AP), followed by US(CC) vs. CBCT and SGRT vs. CBCT. Least agreement between SGRT vs. US(CC).

	SGRT vs. CBCT	US(CC) vs. CBCT	SGRT vs. US(AP)	SGRT vs. US(CC)
Extreme values of differences [mm]	MIN −5.7 MAX 5.9	MIN −6.5 MAX 11.7	MIN −6.0 MAX 6.1	MIN −9.0 MAX 13.0
Wilcoxon signed rank test	significant difference*	n.s.	n.s.	significant difference*
Maloney and Rastogi’s test	PCC −0.25^†^	PCC 0.21^†^	PCC −0.02 (n.s.)	PCC −0.06^†^
PCC	0.53 (CI 0.52–0.55)^†^	0.52 (CI 0.50–0.53)^†^	0.61 (CI 0.60–0.62)^†^	0.44 (CI 0.42–0.46)^†^
	0.61 (CI 0.59–0.63)* *^‡^*	0.54 (CI 0.51–0.56)* *^‡^*	0.64 (CI 0.62–0.66)* *^‡^*	0.47 (CI 0.44–0.49)* *^‡^*
ICC	0.51 (CI 0.49–0.53)^†^	0.49 (CI 0.47–0.51)^†^	0.60 (CI 0.59–0.61)^†^	0.42 (CI 0.4–0.44)^†^
DIBHs with PCC ≥ 0.5*^‡^	67%	76%	80%	70%
DIBHs with PCC ≤ -0.5*^‡^	10%	9%	9%	12%

*p < 0.05, ^†^p < 0.0001, ^‡^DIBHs with residual motion < 2 mm excluded.

Abbreviations: SGRT = surface-guided radiotherapy, CBCT = cone-beam CT, US = ultrasound, CC = craniocaudal, AP = anterior/posterior, MIN = minimum, MAX = maximum, n.s. = not significant, PCC = Pearson's correlation coefficient, ICC = interclass correlation coefficient, CI = confidence interval, DIBH = deep inspiration breath hold.

### 3.3 Detailed analysis per DIBH and per patient

In AP-direction there were more DIBHs with a maximum numerical residual motion <2 mm in US(AP) (75%) than SGRT (65%). In CC-direction the amount of DIBHs with a maximum residual motion <2 mm detected via US(CC) (73%) or CBCT (72%) was comparable. An almost static situation with maximum residual motion of 2 mm in both analyzed motion detection methods was seen in 150/283 (53%) DIBHs for SGRT vs. CBCT, in 181/294 (62%) for US(CC) vs. CBCT, in 185/325 (57%) for SGRT vs. US(AP) and in 180/325 (55%) for SGRT vs. US(CC). Correlation analysis was performed for all DIBHs except those with an almost ideal situation, results can be found in Table 1.

**Fig. 3:**
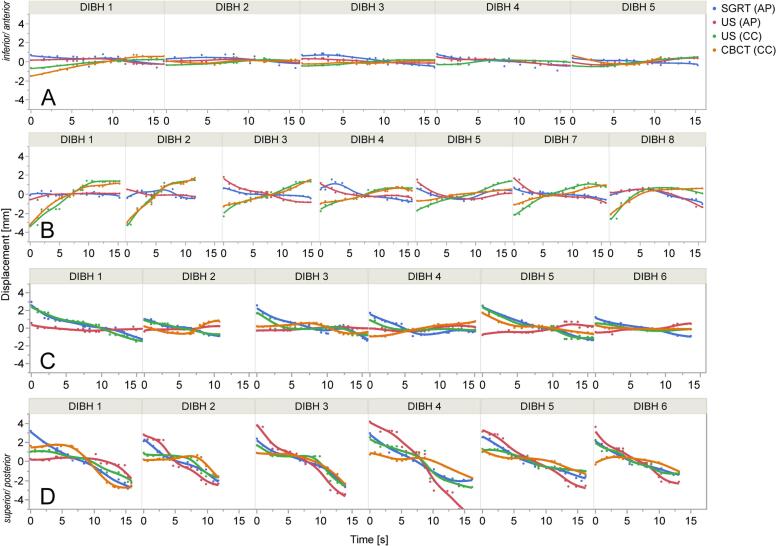
Residual motion curves of 4 fractions from 4 different patients. A) SBRT fraction with 5 DIBHs: Residual motion is consequently <2 mm in all motion detection methods. B) SBRT fraction with 8 DIBHs. Detected motion of SGRT and US(AP) is <2 mm in all DIBHs. CBCT and US(CC) significantly correlate positive (PCC ≥ 0.5, p < 0.05) in all DIBHs with residual motion ≥2 mm (DIBH 1, 2, 3, 7, 8). SGRT and US(CC) show a significant negative correlation in 2 DIBHs (3, 7) and a non-significant negative correlation in 3 DIBHs (1, 2, 8) and 2 DIBHs with a residual motion <2 mm. C) SBRT fraction with 6 DIBHs: All DIBHs of SGRT and US(AP) either show a negative correlation (DIBH 3 and 5 significant, DIBH 1 non-significant) or a residual motion <2 mm. Between US(CC) and CBCT only DIBH 5 shows a significant positive correlation, all other DIBHs are <2 mm or miss data. Between SGRT and US(CC) 3 DIBHs correlate positive significantly (1, 3, 5) while the remaining DIBHs are <2 mm. D) SBRT fraction with 6 DIBHs: Between SGRT and US(AP/CC) all DIBHs show a significant positive correlation. CBCT and US(CC) significantly correlate positive in 4 DIBHs (1, 3, 4, 6) with the other DIBHs showing a residual motion <2 mm.

In 8 out of 12 patients at least 75% of all DIBHs had a residual motion <2 mm in both US(CC) and US(AP). 7 patients in SGRT and 5 patients in CBCT showed a residual motion <2 mm in ≥75%. The individual residual motion curves during DIBH-CBCT of four exemplary SBRT fractions from different patients can be found in Fig. 3.

## 4 Discussion

The aim of this prospective study was to evaluate whether SGRT is an adequate surrogate for monitoring residual intrafractional internal motion during upper abdominal DIBH-SBRT. Detected residual motion of the SGRT-system was compared with detected internal residual motion of a 4D-US and the kV-projections of the DD in DIBH-CBCT as internal motion detection benchmark. Residual motion during DIBH was small in all methods (IQR 0.7–0.9 mm). SGRT(AP) vs. CBCT(CC) and US(CC) vs. CBCT(CC) showed comparable agreement (PCCs 0.53 and 0.52, ICCs 0.51 and 0.49) with slightly higher precision of CBCT(CC). Most agreement was observed for SGRT(AP) vs. US(AP) with largest PCC (0.61) and ICC (0.60), least agreement for SGRT(AP) vs. US(CC) with smallest PCC (0.44) and ICC (0.42).

In the past years different measuring methods for residual intrafractional motion during DIBH for SBRT of abdominal organs have been published: Brown et al. found a mean CC-diaphragm feathering during DIBH in CBCTs of 0.9 mm (25% of all DIBHs >2 mm) [22]. In another study intrafractional shifts were determined by acquiring two CBCTs and comparing the distance of a breathing-dependent structure (IQR 2.8 mm, total range −27 to +30 mm) [23]. With the first clinical implementation of US for DIBH-SBRT we found a residual motion <2 mm (CC) per DIBH in 51% of all DIBHs via US and in 59% of all DIBHs via the DD position in CBCT [16].

In extension to these studies, we used three different intrafractional motion detection methods in the present study simultaneously to determine residual motion during DIBH. With at least 92% of all readings below 2 mm during spirometry induced DIBH and IQR ranging from 0.7 to 0.9 mm residual motion was small and comparable for all detection methods. 65% of all DIBHs showed a residual motion <2 mm in SGRT compared to 75% (US(AP)), 73% (US(CC)) and 72% (CBCT). This may indicate that, in comparison to internal organ motion, SGRT measures larger surface motion especially in the ranges below 2 mm. However, we also measured rare maximal motion up to 1 cm in US(AP) and US(CC). The comparably large maximum residual motion in US could be attributed to a higher sensitivity for internal organ motion in the liver (no DD or surface as surrogate) or simply to the fact that for US the highest number of DIBHs were analyzed with a higher probability for detection of larger motion.

In 8 out of 12 patients at least 75% of all DIBHs had a residual motion <2 mm in US(CC) and US(AP), in SGRT (7 patients) and CBCT (5 patients) even less. A higher residual motion in one patient could not be narrowed down to certain DIBHs or fractions but seemed to be caused by different breathing patterns. With the implementation of individual GTV-PTV margins, soft-tissue based monitoring (US, MRI [13], [14]) or adaptive planning such individual residual motion patterns could be compensated, consecutively sparing normal tissue or ensuring sufficient dose coverage. If using SGRT only, further evaluation of needed treatment margins is necessary to avoid marginal misses on an individual basis. Residual motion data during the planning-CT or the first treatment fraction can be used for PTV margin adaptation during the series (e.g., larger margins for patients with higher residual motion).

Some studies suggested that SGRT can be used as surrogate for internal motion during 4D-CT/CBCT or BH for individual patients [34], [35], [36]. Zeng et al. recently discouraged using SGRT as solo monitoring method and Wang et al. recommended patient-based assessment of internal/external correlation as correlation differed by a high degree for various patients [37], [38]. The AAPM task group states that SGRT may not track the internal target with sufficient accuracy and therefore recommends a synergistic approach in combination with internal imaging [53]. Using multiple data points per DIBH for each method enabled us to continuously supervise the range of movement and record the worst-case scenario of large intrafractional motion. Agreement analysis as well as the percentage of correlating DIBHs showed comparable agreement of internal CC-motion in CBCT with surface AP-motion (SGRT) and internal CC-motion (US). When comparing SGRT with US, SGRT clearly agreed more with US(AP) than US(CC). Least agreement between SGRT and US(CC) may indicate inadequacy of SGRT and diaphragm-dome as surrogates for internal CC-motion: Measuring the diaphragm-dome only offers one-dimensional information and correlation with internal target motion depends highly on target localization in the upper abdomen [54], [55]. Organ filling and peristaltic motion can influence target position additionally to breathing. On that account SGRT, although showing good agreement with CBCT, may not be a sufficient surrogate for target motion in the upper abdomen. Especially during free-breathing soft-tissue-based monitoring (US) assures maximum security as it offers motion information on the target itself. For the DIBH situation, SGRT detected large motion during DIBH in most cases which could reliably trigger beam interruption.

At least 92% of all readings showed a residual motion <2 mm whereas only 65%–75% of all DIBHs showed residual motion <2 mm. Although most readings per DIBH were <2 mm there were many DIBHs with single readings exceeding this range. Additionally, residual motion of 2 mm included negative and positive deviation around the mean value per DIBH, resulting in an absolute range of 4 mm. The measuring errors of each motion detection method (US 1.4 ± 1.6 mm, SGRT <1 mm, spirometry system <2 mm, CBCT 2.2 mm) were generously encompassed with those margins, making it more probable to detect actual changes in residual motion by reducing the analysis to DIBHs with residual motion ≥2 mm in both analyzed methods. Nevertheless, our results with and without DIBHs with residual motion <2 mm are comparable. This may be an indicator that despite the possible evaluation of measuring errors during DIBH the underlying outcome stays the same. In some DIBHs (9–12%) a significant negative correlation between motion detection methods was found. Negative correlations could be caused by a detection error of one method, paradox breathing or arbitrary patient motion during DIBH.

Our findings suggest that SGRT alone is not a sufficient surrogate for residual internal CC-motion in all patients as some high velocity motion could not be detected. Least agreement between SGRT and US(CC) may indicate inadequacy of SGRT and diaphragm-dome as surrogates for internal CC-motion whereas direct soft-tissue based monitoring seems to offer maximum security. Additionally, breathing patterns can be individual with a necessity for compensation. Potential triggering limits with any of the examined methods should be accounted for during PTV margin design. Limitations of this study are the small number of patients and the relatively high amount of missing data. Furthermore, detections were made during the pre-treatment CBCT and not directly during beam delivery, leaving the possibility of the liver drifting with time on the couch. Clinical experience with the US for abdominal SBRT is limited leaving questions like target deformation caused by the US probe, radiation tolerance of the probe or CBCT artifacts yet unanswered, however it is subject of evaluation in ongoing research.

## Funding

Parts of these studies were supported by a research grant from Elekta AB, Sweden. We thank Elekta AB for providing the ultrasound system as a test-version in the framework of a research agreement. For the publication fee we acknowledge financial support by Deutsche Forschungsgemeinschaft within the funding programme „Open Access Publikationskosten“ as well as by Heidelberg University.

## Declaration of Competing Interest

The authors declare the following financial interests/personal relationships which may be considered as potential competing interests: Frank A. Giordano reports honoraria, research grants and/or travel support from Carl Zeiss Meditec AG, TME Pharma AG, Guerbet SA, Cureteq AG, Bristol-Myers Squibb, AstraZeneca GmbH, FoMF GmbH, MEDAC GmbH, Elsevier GmbH and stock/ownership from TME Pharma AG and Implacit GmbH. Daniel Buergy reports personal fees from Siemens AG, NB Capital Research GmbH, NB Capital ApS, PharmaMar GmbH and b.e. Imaging GmbH outside the submitted work. Lena Kaestner, Lara Streb, Svetlana Hetjens, Dwi S.K. Sihono, Jens Fleckenstein, Iris Kalisch, Miriam Eckl, Frank Lohr, Florian Stieler and Judit Boda-Heggemann have nothing to declare.
